# Luminescence properties and energy transfer of Nd^3+^- Er^3+^/ Nd^3+^-Pr^3+^ co-doped LFP glasses system

**DOI:** 10.1016/j.heliyon.2023.e21114

**Published:** 2023-10-23

**Authors:** Juniastel Rajagukguk, Jonny H. Panggabean, C.S. Sarumaha, P. Kanjanaboos, N. Phuphathanaphong, S. Kothan, J. Kaewkhao, Mitra Djamal

**Affiliations:** a Department of Physics, Faculty of Mathematics and Natural Sciences, Universitas Negeri Medan, Medan 20221, Indonesia; b Physics Program, Faculty of Science and Technology, Nakhon Pathom Rajabhat University, Nakhon Pathom 73000, Thailand; c Center of Excellence in Glass Technology and Materials Science (CEGM), Nakhon Pathom Rajabhat University, Nakhon Pathom 73000, Thailand; d School of Materials Science and Innovation, Faculty of Science, Mahidol University, Nakhon Pathom 73170, Thailand; e Center of Radiation Research and Medical Imaging, Department of Radiologic Technology, Faculty of Associated Medical Sciences, Chiang Mai University, Chiang Mai, 50200, Thailand; f Department of Physics, Faculty of Mathematics and Natural Science, Institut Teknologi Bandung, Jl. Ganesha No. 10, Bandung 46123, Indonesia

**Keywords:** Luminescence, FWHM, Energy transfer, Quantum yields

## Abstract

The motivation for this research is that the emission spectra using directly pumped laser diodes have not yet been found. We want to explore the luminescence properties of a co-doped laser material utilizing a diode laser as an optical pump. The research method used standard melt-quench and was stimulated by a laser diode (808 and 980 nm). The double doped of Nd^3+^- Er^3+^/Nd^3+^-Pr^3+^ ions with glasses system of lithium-fluorophosphate (LFP) had a strong band emission at 1056 nm, which transitioned at ^4^F_3/2_ → ^4^I_11/2_ and showed a drop in intensity from co-doping with Er^3+^ and Pr^3+^ ions. The fluorescence width at half maximum (FWHM) of the glasses is calculated to identify whether the sample may be used as a laser application. The FWHM values are found to be 22–28 nm. Decay time values were shown to decrease with increasing concentrations of Er^3+^ and Pr^3+^ ions and were used for energy transfer calculations. The Quantum Yields (QYs), efficiency in the transfer of energy and the possibility transfer energy were measured and calculated that confirm the possibility of energy transfer from Nd^3+^ to Er^3+^ and Pr^3+^ ions. Since, the emission spectrum at 1535 nm was found, this is a good reason for it to be used as an optical device.

## 1 Introduction

Luminescence is a phenomenon that has fascinated scientists for centuries and has numerous practical applications. In recent years, the study of glass material doped single rare-earth ion has studied from structural, thermal, and electrical characteristics for photonic and laser applications was studied [[1], [2], [3]]. The co-doping process modifies the luminescence properties of the glass system in various ways. For instance, it can enhance the luminescence intensity and broaden the emission band [4,5]. This is because the co-doping process introduces additional energy levels that facilitate energy transfer between different ions. Moreover, the co-doping process can affect the lifetime of excited states and emission spectra, which are prominent factors in practical applications. The optical, structural, luminescence, and energy transfer examination covered some of the prior research on the co-doping of Nd^3+^ with other RE ions [[6], [7], [8], [9], [10]]. Eu^3+^/Nd^3+^ co-doped borosilicate glass was used in luminescence application with effective sensitization of Eu^3+^ ions [11], while Nd^3+^/Yb^3+^ co-doped silicate glass was examined for energy transfer by temperature dependence [12]. Tellurite glass that is co-doped with Nd^3+^ and Tm^3+^ has its broadband near-infrared luminescence property examined [13]. H. Yin et al. [14] have reported regarding fluoride halide glass co-doped with Nd^3+^ and Ho^3+^ ions, demonstrating tunability across several wavelengths, including 1064 nm, 2.0 μm, and 2.9 μm. Nd^3+^/Yb^3+^ in fluorophosphate glasses for optical thermometry in near-infrared has been reported [15]. According to K. Sriramulu et al. [16], Nd^3+^ and Yb^3+^ co-doped glass can produce efficient solar energy concentrators and lasers. However, Yan-Jie Song et al. reported that the back energy transfer from Yb^3+^ to Nd^3+^ was negligible [17]. This proves that the study of energy transfer from Nd^3+^ to other RE^3+^ ions requires a lot of understanding and study. Choosing the proper host material is essential for efficient energy transfer since the host material significantly affects the sensitized luminescence. As explained in our earlier research, lithium fluorophosphate was our material of choice for the present glass [18,19]. The energy transfer mechanisms involved in the Nd^3+^-Er^3+^/Nd^3+^-Pr^3+^ co-doped LFP glasses system are complex but fascinating. When excited, both Nd^3+^ and Er^3+^ ions emit light at different wavelengths due to their unique energy levels. The co-doping process affects the energy transfer between these ions, leading to attractive phenomena such as up-conversion and down-conversion luminescence [5]. When the Nd^3+^ ions absorb low-energy photons and then transfer them to the Er^3+^ ions through a non-radiative energy transfer process, up-conversion luminescence takes place. This causes the Er^3+^ ions to emit light at higher energies than they would ordinarily be capable of. However, down-conversion luminescence happens when high-energy photons enter the Er^3+^ ions and then move to the Nd^3+^ ions. This leads to the emission of lower-energy light than would be expected from the Er^3+^ ions. By understanding the luminescence properties and energy transfer mechanisms of Nd^3+^-Er^3+^/Nd^3+^-Pr^3+^ co-doped LFP glasses systems, we can unlock new possibilities for lasers, optical amplifiers, and sensors applications. This research can revolutionize the field of optics and lead to exciting new developments.

In the present research, the Nd^3+^-Er^3+^/Nd^3+^-Pr^3+^ co-doped LFP were characterized by near-infrared luminescence (NIR) with laser diode source and the efficiency energy transfer with Dexter and Föster calculation.

## 2 Experiments

**Fig. 1:**
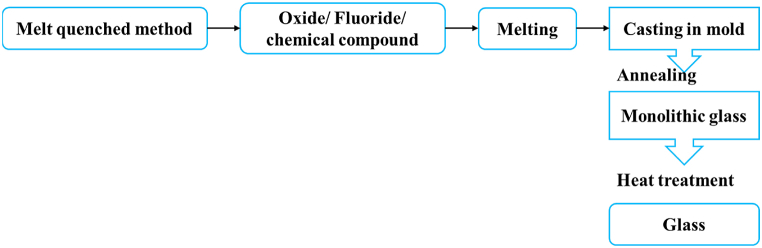
The scheme of the melt-quench technique.

The glass samples under investigation consist of a co-doping of Nd^3+^-Er^3+^/Nd^3+^-Pr^3+^ ions with a composition shown in Table 1. The fabrication process employed in this work is the melt-quenching technique, shown in Fig. 1. For each sample, the reagents with a purity of 99.99 % were weighed and combined in an alumina crucible in powder form. All of the samples were prepared using the same methodology that called as melted-quenched technique which outlined in our previous publication [18,19]. Characterization is assessed after the process of cutting the glass and ensuring its appropriate dimensions. The luminescence spectra and decay time of Near-Infrared (NIR) were measured using the (QM)-300 spectrofluorometer manufactured by PTI-Horiba using laser diode source (808 and 980 nm). The energy transfer parameters such as the efficiency and probability were calculated using the Dexter and Föster formula.

**Table 1:** Compositions (mol%) of Er^3+^- Nd^3+^/Pr^3+^-Nd^3+^ double doped LFP glasses system.

Label Glass	Li_2_O	CaF_2_	P_2_O_5_	Nd_2_O_3_	Er_2_O_3_	Ref.
Nd-Pure	0.2	0.14	0.65	0.01	0	[15]
NdEr2	0.2	0.14	0.645	0.01	0.005	
NdEr3	0.2	0.14	0.64	0.01	0.01	
NdEr4	0.2	0.14	0.635	0.01	0.015	
NdEr5	0.2	0.14	0.63	0.01	0.02	
**Label Glass**	**Li**_**2**_**O**	**CaF**_**2**_	**P**_**2**_**O**_**5**_	**Nd**_**2**_**O**_**3**_	**Pr**_**2**_**O**_**3**_	
Nd-Pure	0.2	0.14	0.65	0.01	0	[16]
NdEr2	0.2	0.14	0.645	0.01	0.005	
NdEr3	0.2	0.14	0.64	0.01	0.01	
NdEr4	0.2	0.14	0.635	0.01	0.015	
NdEr5	0.2	0.14	0.63	0.01	0.02

## 3 Results and discussion

**Fig. 2:**
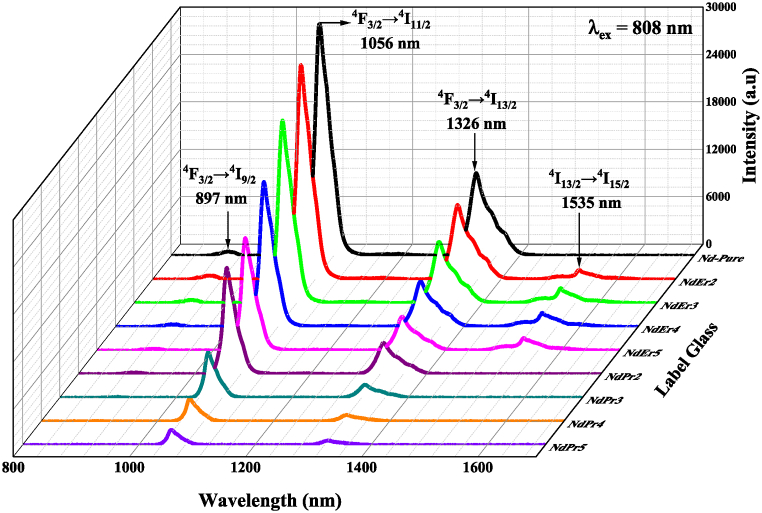
Emission spectra (excited by a laser diode at 808 nm) of Er^3+^- Nd^3+^/Pr^3+^-Nd^3+^ double doped LFP glasses.

The emission spectra were excited by 808 nm of laser diode as shown in Fig. 2. The band emission prominent of Nd^3+^ was 1056 nm which the transition at ^4^F_3/2_ → ^4^I_11/2_ and show intensity decrease was co-doped with Er^3+^ and Pr^3+^. But the most striking decrease occurred when doped with Pr^3+^ ions. The Nd^3+^ ions are designed as donors, whereas the ions, namely Er^3+^ and Pr^3+^ ions, play the role of acceptors responsible for depopulating donor levels. As a result, we noticed that the luminescence intensity is attenuated after the initial addition of Er^3+^. However, further addition of Er^3+^ ions improved the luminescence intensity at 1535 with the transition of ^4^I_13/2_ → ^4^I_15/2_ and quench at NdEr4 (1.5 mol% of Er^3+^ ions). This behavior shows that the effectiveness of energy transfer between donor-acceptor ions depends on the overlap between the donor's emission and the activator's absorption, which in turn impacts whether luminescence intensity is increased or decreased. The experiments [18,19] demonstrated that a rise in the density of the glass sample led to a corresponding decrease in the emission intensity of the co-doping sample. However, because of the wavelength overlap with the Nd^3+^ ion, the peak of the Pr^3+^ ion is imperceptible. The band of Pr^3+^ ions is located around the 1040 nm region, as our prior research stated [19]. This demonstrates that band of Pr^3+^ is unseen when stimulated directly by Nd^3+^ ions.

**Fig. 3:**
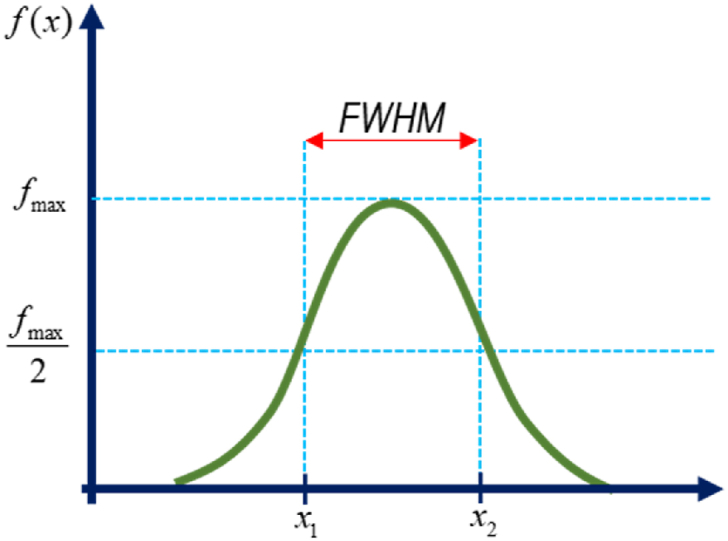
The fluorescence width at half maximum (FWHM).

After exciting with an 808 nm laser diode, we observed a strong NIR emission at 1056 nm, with a fluorescence width at half maximum (FWHM) ranging from 22 to 28 nm, as detailed in Table 2. FWHM represents the spectral curve's width, determined by measuring the distance between two points on the y-axis where the amplitude is half of its maximum value (Fig. 3) [20]. The line-width of a single-frequency laser corresponds to the FWHM of its optical spectrum. To be more specific, it quantifies the power spectral density of the emitted electric field concerning wavenumber, frequency or wavelength. The laser's narrow FWHM is intricately connected to its temporal coherence. The utilization of lasers with exceptionally narrow linewidth is imperative for a multitude of applications. These include serving as light optical sources for diverse types of fiber sensors, facilitating spectroscopy (such as L-I-D-A-R sensor), enabling coherent optical fiber communications, and supporting atomic trapping and cooling processes. For instance, FWHM of 10 nm or fewer are regarded as narrowband and frequently employed for chemical detection and laser cleaning. Machine vision applications frequently use FWHM of 25–50 nm; FHWM greater than 50 nm are regarded as broadband and are frequently used in fluorescence microscopy applications [21]. Z. Zhou et al. reported FWHM under excitation 480 nm with single Pr^3+^ (60 nm), single Nd^3+^ (30 nm) and co-doped (66 nm) [22]. Our earlier research revealed the FWHM values for the co-doped sample with excitation 481 nm were 26 nm (NdPr3) and 38 nm (NdPr4) [19]. According to some published research, the FWHM in Nd^3+^ doped lithium fluoro-borate glasses with various modifier oxides demonstrates close to 49 nm under excitation at 808 nm [23] and 29 nm for commercially available phosphate laser glasses [24]. This report leads to the emission spectra being wider than our present work. However, M. Guo et al. reported that their Nd^3+^-doped ALP sample has FWHM in the range of 23–24.4 which is smaller than our sample which was co-doped with Nd^3+^-Er^3+^ ions [25]. Additionally, it demonstrates that our glass sample exhibits a lower value when excited by an 808 nm laser diode than when excited by a 481 nm laser, making it more appropriate for laser applications.

**Table 2:** FWHM (^4^F_3/2_ → ^4^I_11/2_ transition) of Er^3+^- Nd^3+^/Pr^3+^-Nd^3+^ double doped LFP glasses.

Samples	FWHM	Exc. Sources	Ref.
Nd-Pure	28 nm	808 nm	Present work
NdEr2	27 nm	808 nm	Present work
NdEr3	27 nm	808 nm	Present work
NdEr4	26 nm	808 nm	Present work
NdEr5	25 nm	808 nm	Present work
NdPr2	24 nm	808 nm	Present work
NdPr3	23 nm	808 nm	Present work
NdPr4	23 nm	808 nm	Present work
NdPr5	22 nm	808 nm	Present work
NdPr3	26 nm	481 nm	[19]
NdPr4	38 nm	481 nm	[19]
Single Pr^3+^	60 nm	480 nm	[22]
Single Nd^3+^	30 nm	480 nm	[22]
Co-doped Nd^3+^-Pr^3+^	66 nm	480 nm	[22]
Nd^3+^: LFB	49 nm	808 nm	[23]
APG-t	29 nm	808 nm	[24]
Nd^3+^-doped ALP	23–24.4 nm	808 nm	[25]

Quantum yields (QYs), which measure a material's efficiency by comparing the emitted photons number to the absorbed photons number, are defined as the ratio [26,27]. Utilizing a xenon lamp as the source of excitation and a Horiba FluoroMax 4+ spectrofluorometer with an integrating sphere (integration time of 1.0 s, excitation of 444 nm, excitation slit of 2.4 nm, measurement of the wavelength emitted between 450 and 750 nm, and emission slit of 3 nm) [28], the QYs measurements were conducted. The following equation (Eq. 1) can be used to compute the fluorescence QYs [29]:

[1] $$\eta =\frac{N_{e}}{N_{a}}=\frac{\int L_{s}}{\int E_{R}-\int E_{S}}$$

By dividing the total amount of photons released by the total amount of photons absorbed by the sample, the QY is computed. *N*_*e*_ and *N*_*a*_ are the sample's respective photon emission and absorption rates. To figure out how many photons were released, one can integrate the luminescence emission spectrum (*L*_*S*_) of the material. By omitting photons that pass through but do not enter the sample, the variance in their integrations can signify the quantity of photons absorbed by the sample. *E*_*R*_ and *E*_*S*_ stand for excitation spectra without and with the sample inside the sphere, respectively. The sphere was used to gather all of the spectra. Table 3 also summarizes the QYs values for the Nd^3+^-Pr^3+^ glasses as determined by an integrating sphere. D. Ding et al. reported a reduction in visible QYs (5 %–0.9 %) when adding Yb^3+^ concentration to the glass [29]. Although only 1.24 %–0.62 %, our results from this report can be considered reasonable [29]. The presence of a decrease in QYs allows for energy transfer between Nd^3+^ and Pr^3+^ ions.

**Table 3:** Quantum yields (QYs) of Pr^3+^-Nd^3+^ double doped LFP glasses.

Samples	Quantum Yield (%)
NdPr2	1.24
NdPr3	0.89
NdPr4	0.61
NdPr5	0.62

**Fig. 4:**
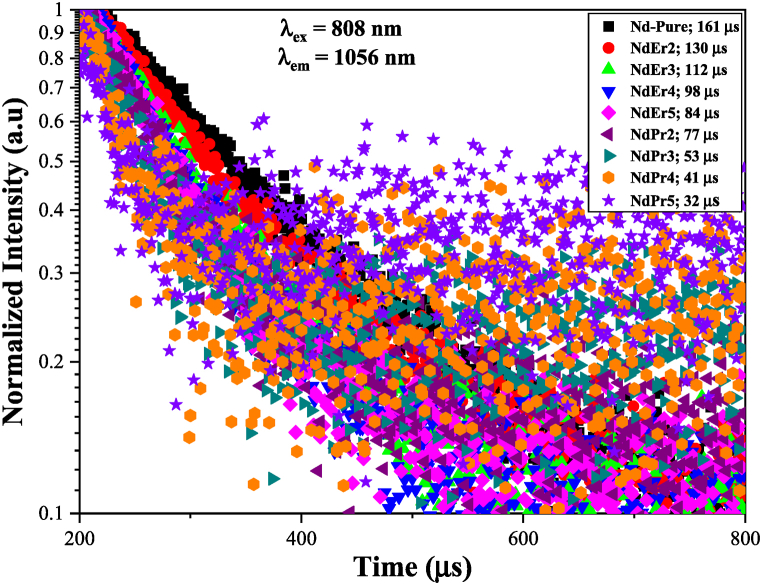
Decay time spectra (excited by a laser diode at 808 nm) of Er^3+^- Nd^3+^/Pr^3+^-Nd^3+^ double doped LFP glasses.

The decay curves of the Nd^3+^: ^4^F_3/2_ level were measured and are displayed in Fig. 4 in order to support the interaction between Nd^3+^- Er^3+^/Nd^3+^-Pr^3+^ in LFP glasses. The decay period of Nd^3+^: ^4^F_3/2_ dropped from 161 s to 32 μs from Nd-pure to Nd co-doping. The shortening of the decay time [5] served as evidence of the mechanism of energy transfer involving ions like Nd^3+^-Er^3+^ and Nd^3+^-Pr^3+^. The energy transfer by level diagram was reported in our earlier work [18,19]. Using lifetime change, the Dexter and Föster formula (Eq. 2-3) can be used to quickly determine the effectiveness and likelihood (probability) of energy transfer from Nd^3+^ to Er^3+^/Pr^3+^ ions [[30], [31], [32]]:

[2] $$\eta_{ET\left(Nd\to Er/\mathrm{Pr}\right)}=1-\left(\frac{\tau_{Nd\_co-doped}}{\tau_{Nd-pure}}\right)$$

In the terms of lifetime, the energy transfer probability can be expressed by Refs. [33,34]:

[3] $$P_{\left(Nd\to Er/\mathrm{Pr}\right)}=\frac{1}{\tau_{Nd\_co-doped}}-\frac{1}{\tau_{Nd-pure}}$$

where *τ*_*Nd_co-doped*_ is the sensitizer's lifetime in the presence of activators (Er^3+^/Pr^3+^) and *τ*_*Nd-pure*_ is intrinsic decay time of sensitizer (Nd^3+^) in the absence of activators. The current analysis provides clear proof of energy transfer occurring from Nd^3+^ to Er^3+^ as well as from Nd^3+^ to Pr^3+^ within the LFP glass system. The data for lifetime (τ μs), probability of energy transfer (P x 10^3^ S^−1^), and efficiency (η_ET_%) are summarized in Table 4.

**Table 4:** Some parameters of energy transfer for Er^3+^- Nd^3+^/Pr^3+^-Nd^3+^ double doped LFP glasses.

Samples	Nd^3+^ (mol%)	Er^3+^ (mol%)	Lifetime of Nd^3+^ (τ μs) ± 0.1	Lifetime of Er^3+^ (τ μs) ± 0.1	(η_ET_%) ± 1	(*P*x10^3^ ms^-1^) ± 0.001
Nd-Pure	1.0	0	161	–	–	–
NdEr2	1.0	0.5	130	378	19	0.001
NdEr3	1.0	1.0	112	392	30	0.003
NdEr4	1.0	1.5	98	216	39	0.004
NdEr5	1.0	2.0	84	238	48	0.006
**Samples**	**Nd**^**3+**^**(mol%)**	**Pr**^**3+**^**(mol%)**	**Lifetime of Nd**^**3+**^**(τ μs)** **± 0**.**1**		**(η**_**ET**_**%) ± 1**	**(*P*x10**^**3**^ ms^**-1**^**) ± 0.001**
NdPr2	1.0	0.5	77		52	0.007
NdPr3	1.0	1.0	53		67	0.013
NdPr4	1.0	1.5	41		75	0.018
NdPr5	1.0	2.0	32		80	0.025

**Fig. 5:**
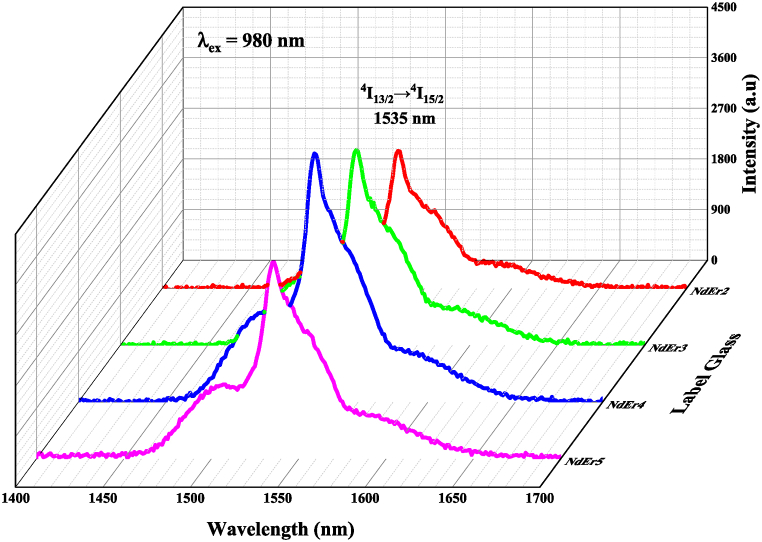
Emission spectra of Er^3+^- Nd^3+^ double doped LFP glasses when excited by a laser diode at 980 nm.

**Fig. 6:**
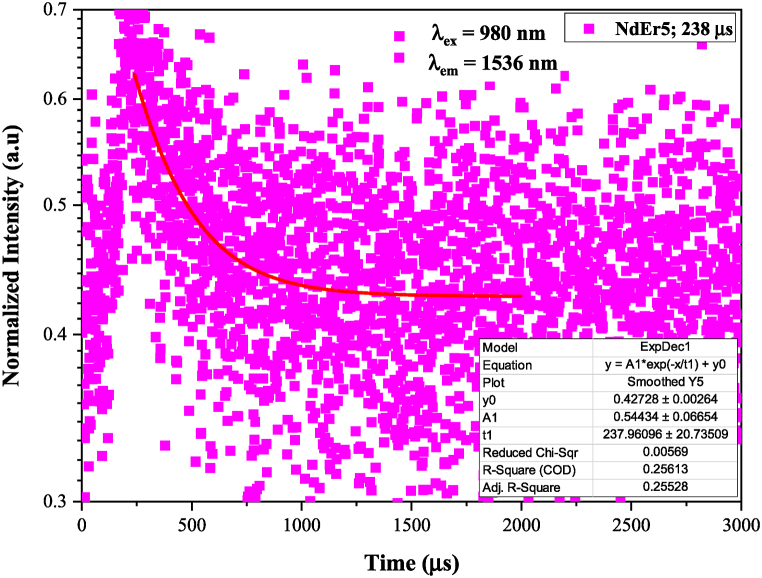
Decay time spectra (excited by a laser diode at 980 nm) of Er^3+^- Nd^3+^ double doped LFP glasses.

Fig. 5 displays the laser diode's 980 nm-excited emission spectrum. The band emission of Er^3+^ had a wavelength of 1535 nm and a transition at ^4^I_13/2_ → ^4^I_15/2_. Its intensity increased up to 1.5 mol% of Er^3+^ ions but decreased when 2 mol% of Er^3+^ ions were added. The trend of emission intensity is the same as the results we previously reported, but the Er^3+^ spectrum in this work is more clearly visible than previously only the noise spectrum [18]. The decay time of Er^3+^ was excited at 980 nm and emit at 1535 nm as shown in Fig. 6. The value of decay time was 378 μs (NdEr2), 392 μs (NdEr3), 216 μs (NdEr4) and 238 μs (NdEr5).

## 4 Conclusions

In summary, the Nd^3+^- Er^3+^/Nd^3+^-Pr^3+^ co-doped LFP glasses system was stimulated by an 808 nm laser diode. Nd^3+^ had a strong band emission at 1056 nm, which transitioned at ^4^F_3/2_ → ^4^I_11/2_ and showed a drop in intensity from co-doping with Er^3+^ and Pr^3+^ ions. However, further addition of Er^3+^ ions can be improved the luminescent intensity at 1535 nm with the transition of ^4^I_13/2_ → ^4^I_15/2_ and quench at NdEr4 (1.5 mol% of Er^3+^ ions). The FWHM of the glasses is calculated to identify whether the sample may be used as a laser application. The FWHM values are found to be 22–28 nm. It has been demonstrated that the value FWHM of glass stimulated by an 808 nm laser diode is lower than that of glass excited by a xenon lamp at 481 nm, making the former more suitable for laser applications. The QYs, transfer energy of efficiency and the probability were measured and calculated to verify the potential for energy transfer to occur from Nd^3+^ to Er^3+^ and Pr^3+^ ions. These samples are great for study in luminescence, but more development is needed for practical use. This sample was significantly hygroscopic at room temperature, highlighting the importance of careful material selection.

## Data availability statement

Data included in article/supplementary material/referenced in article.

## CRediT authorship contribution statement

**Juniastel Rajagukguk:** Conceptualization, Data curation, Funding acquisition, Project administration, Resources, Writing – original draft, Writing – review & editing. **Jonny H. Panggabean:** Formal analysis, Visualization, Writing – review & editing. **C.S. Sarumaha:** Data curation, Formal analysis, Software, Visualization, Writing – original draft. **P. Kanjanaboos:** Data curation, Investigation, Visualization. **N. Phuphathanaphong:** Data curation, Visualization. **S. Kothan:** Data curation, Formal analysis, Writing – review & editing. **J. Kaewkhao:** Conceptualization, Data curation, Formal analysis, Investigation, Validation. **Mitra Djamal:** Formal analysis, Supervision, Visualization.

## Declaration of competing interest

The authors declare that they have no known competing financial interests or personal relationships that could have appeared to influence the work reported in this paper.
