# Supplementary material for: Luminescence properties and energy transfer of Nd3+- Er3+/ Nd3+-Pr3+ co-doped LFP glasses system
Source: Heliyon. 2023 Oct 23;9(11):e21114. doi: 10.1016/j.heliyon.2023.e21114 (PMC10628660; doi:10.1016/j.heliyon.2023.e21114)
Supplement: Multimedia component 1 [file mmc1.docx]

**Supplementary data figures Journal**

**Fig. 1.** The scheme of the melt-quench technique

**Fig. 2.** Emission spectra (excited by a laser diode at 808 nm) of Er^3+^- Nd^3+^/ Pr^3+^-Nd^3+^ double doped LFP glasses

**Fig. 3.** The fluorescence width at half maximum (FWHM)

**Fig. 4.** Decay time spectra (excited by a laser diode at 808 nm) of Er^3+^- Nd^3+^/ Pr^3+^-Nd^3+^ double doped LFP glasses

**Fig. 5.** Emission spectra of Er^3+^- Nd^3+^ double doped LFP glasses when excited by a laser diode at 980 nm

**Fig. 6.** Decay time spectra (excited by a laser diode at 980 nm) of Er^3+^- Nd^3+^ double doped LFP glasses


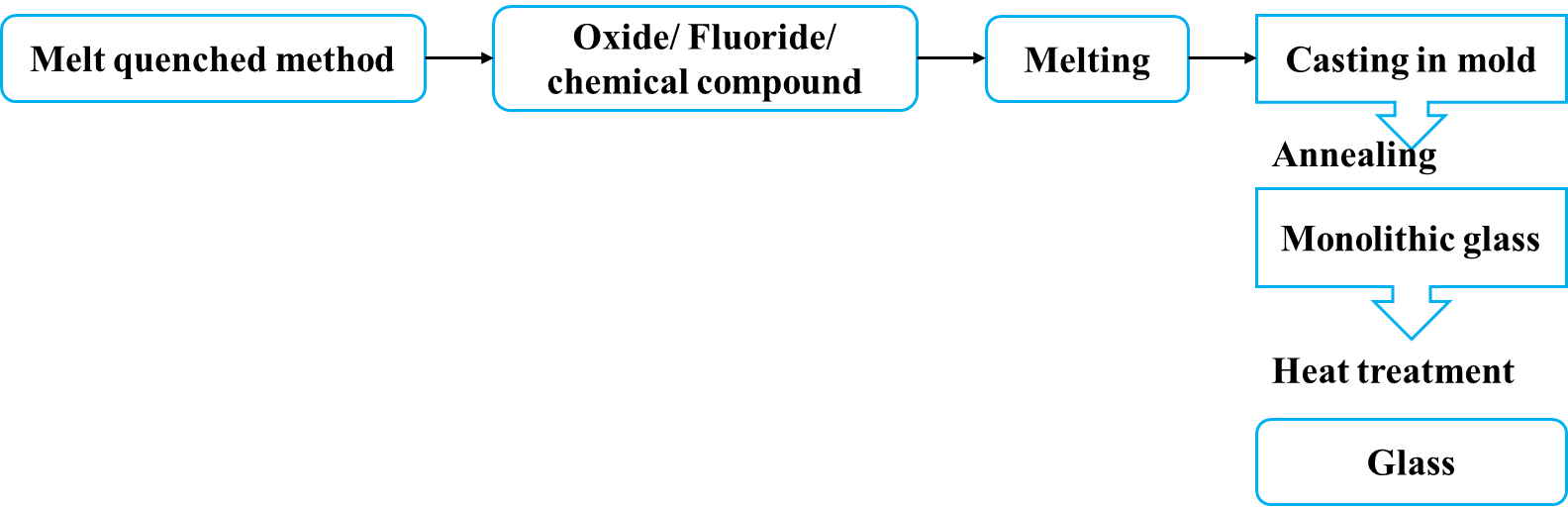


**Figure 1**

**Figure 2**


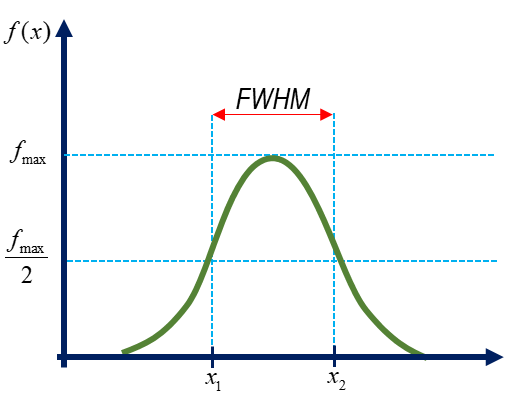


**Figure 3**

**Figure 4**

**Figure 5**

**Figure 6**
