# Supplementary material for: Luminescence properties and energy transfer of Nd3+- Er3+/ Nd3+-Pr3+ co-doped LFP glasses system
Source: Heliyon. 2023 Oct 23;9(11):e21114. doi: 10.1016/j.heliyon.2023.e21114 (PMC10628660; doi:10.1016/j.heliyon.2023.e21114)
Supplement: Multimedia component 2 [file mmc2.docx]

**Supplementary data table Journal**

**Table 1.** Compositions (mol%) of Er^3+^- Nd^3+^/ Pr^3+^-Nd^3+^ double doped LFP glasses system

**Table 2.** FWHM (^4^F_3/2_→^4^I_11/2_ transition) of Er^3+^- Nd^3+^/ Pr^3+^-Nd^3+^ double doped LFP glasses

**Table 3.** Quantum yields (QYs) of Pr^3+^-Nd^3+^ double doped LFP glasses

**Table 4.** Some parameters of energy transfer for Er^3+^- Nd^3+^/ Pr^3+^-Nd^3+^ double doped LFP glasses

**Table 1**

| **Label Glass** | **Li_2_O_3_** | **CaF_2_** | **P_2_O_5_** | **Nd_2_O_3_** | **Er_2_O_3_** | **Ref.** |
| --- | --- | --- | --- | --- | --- | --- |
| Nd-Pure | 0.2 | 0.14 | 0.65 | 0.01 | 0 | [15] |
| NdEr2 | 0.2 | 0.14 | 0.645 | 0.01 | 0.005 |  |
| NdEr3 | 0.2 | 0.14 | 0.64 | 0.01 | 0.01 |  |
| NdEr4 | 0.2 | 0.14 | 0.635 | 0.01 | 0.015 |  |
| NdEr5 | 0.2 | 0.14 | 0.63 | 0.01 | 0.02 |  |
| **Label Glass** | **Li_2_O_3_** | **CaF_2_** | **P_2_O_5_** | **Nd_2_O_3_** | **Pr_2_O_3_** |  |
| Nd-Pure | 0.2 | 0.14 | 0.65 | 0.01 | 0 | [16] |
| NdEr2 | 0.2 | 0.14 | 0.645 | 0.01 | 0.005 |  |
| NdEr3 | 0.2 | 0.14 | 0.64 | 0.01 | 0.01 |  |
| NdEr4 | 0.2 | 0.14 | 0.635 | 0.01 | 0.015 |  |
| NdEr5 | 0.2 | 0.14 | 0.63 | 0.01 | 0.02 |  |

**Table 2**

| **Samples** | **FWHM** | **Exc. Sources** | **Ref.** |
| --- | --- | --- | --- |
| Nd-Pure | 28 nm | 808 nm | Present work |
| NdEr2 | 27 nm | 808 nm | Present work |
| NdEr3 | 27 nm | 808 nm | Present work |
| NdEr4 | 26 nm | 808 nm | Present work |
| NdEr5 | 25 nm | 808 nm | Present work |
| NdPr2 | 24 nm | 808 nm | Present work |
| NdPr3 | 23 nm | 808 nm | Present work |
| NdPr4 | 23 nm | 808 nm | Present work |
| NdPr5 | 22 nm | 808 nm | Present work |
| NdPr3 | 26 nm | 481 nm | [19] |
| NdPr4 | 38 nm | 481 nm | [19] |
| Single Pr^3+^ | 60 nm | 480 nm | [22] |
| Single Nd^3+^ | 30 nm | 480 nm | [22] |
| Co-doped Nd^3+^-Pr^3+^ | 66 nm | 480 nm | [22] |
| Nd^3+^: LFB | 49 nm | 808 nm | [23] |
| APG-t | 29 nm | 808 nm | [24] |
| Nd^3+^-doped ALP | 23-24.4 nm | 808 nm | [25] |

**Table 3**

| **Samples** | **Quantum Yield (%)** |
| --- | --- |
| NdPr2 | 1.24 |
| NdPr3 | 0.89 |
| NdPr4 | 0.61 |
| NdPr5 | 0.62 |

**Table 4**

| **Samples** | **Nd^3+^ (mol%)** | **Er^3+^ (mol%)** | **Lifetime of Nd^3+^ (τ μs) ± 0.1** | **Lifetime of Er^3+^ (τ μs) ± 0.1** | **(η_ET_%) ± 1** | **(*P*x10^3^ S^-1^) ± 0.001** |
| --- | --- | --- | --- | --- | --- | --- |
| Nd-Pure | 1.0 | 0 | 161 | - | - | - |
| NdEr2 | 1.0 | 0.5 | 130 | 378 | 19 | 0.001 |
| NdEr3 | 1.0 | 1.0 | 112 | 392 | 30 | 0.003 |
| NdEr4 | 1.0 | 1.5 | 98 | 216 | 39 | 0.004 |
| NdEr5 | 1.0 | 2.0 | 84 | 238 | 48 | 0.006 |
| **Samples** | **Nd^3+^ (mol%)** | **Pr^3+^ (mol%)** | **Lifetime of Nd^3+^ (τ μs)  ± 0.1** | | **(η_ET_%) ± 1** | **(*P*x10^3^ S^-1^) ± 0.001** |
| NdPr2 | 1.0 | 0.5 | 77 | | 52 | 0.007 |
| NdPr3 | 1.0 | 1.0 | 53 | | 67 | 0.013 |
| NdPr4 | 1.0 | 1.5 | 41 | | 75 | 0.018 |
| NdPr5 | 1.0 | 2.0 | 32 | | 80 | 0.025 |
